# The Rapid Arterial oCclusion Evaluation (RACE) scale accuracy for diagnosis of acute ischemic stroke in emergency department – A multicenter study

**DOI:** 10.1186/s12873-023-00825-7

**Published:** 2023-05-24

**Authors:** Hosein Rafiemanesh, Negin Barikro, Somayeh Karimi, Mehran Sotoodehnia, Alireza Jalali, Alireza Baratloo

**Affiliations:** 1 Non-communicable Diseases Research Center, Alborz University of Medical Sciences, Karaj, Iran; 2 Department of Epidemiology and Biostatistics, School of Public Health, Alborz University of Medical Sciences, Karaj, Iran; 3 Department of Emergency Medicine, Sina Hospital, Tehran University of Medical Sciences, Tehran, Iran

**Keywords:** Decision support techniques, Emergency service, hospital, Stroke

## Abstract

**Objective:**

It seems that the available data on performance of the Rapid Arterial oCclusion Evaluation (RACE) as a prehospital stroke scale for differentiating all AIS cases, not only large vessel occlusion (LVO), from the stroke mimics is lacking. As a result, we intend to evaluate the accuracy of the RACE criteria in diagnosing of AIS in patients transferred to the emergency department (ED).

**Method:**

The present study was a diagnostic accuracy cross-sectional study during 2021 in Iran. The study population consist of all suspected acute ischemic stroke (AIS) patients who transferred to the ED by emergency medical services (EMS). A 3-part checklist consisting of the basic and demographic information of the patients, items related to the RACE scale, and the final diagnosis of the patients based on interpretation of patients’ brain MRI was used for data collection. All data were entered in Stata 14 software. We used the ROC analysis to evaluate the diagnostic power of the test.

**Result:**

In this study, data from 805 patients with the mean age of 66.9 ± 13.9 years were studied of whom 57.5% were males. Of all the patients suspected of stroke who transferred to the ED, 562 (69.8%) had a definite final diagnosis of AIS. The sensitivity and specificity of the RACE scale for the recommended cut-off point (score ≥ 5) were 50.18% and 92.18%, respectively. According to the Youden J index, the best cut-off point for this tool for differentiating AIS cases was a score > 2, at which sensitivity and specificity were 74.73% and 87.65%, respectively.

**Conclusion:**

It seems that, the RACE scale is an accurate diagnostic tool to detect and screen AIS patients in ED, Of course, not at the previously suggested cut-off point (score ≥ 5), but at the score > 2.

**Supplementary Information:**

The online version contains supplementary material available at 10.1186/s12873-023-00825-7.

## Introduction

Stroke is one of the most common leading cause of both disability and death worldwide, and more than 80% of stroke cases are ischemic types globally [1]. Despite of introduction of intravenous thrombolytic therapy and also mechanical thrombectomy for curing acute ischemic stroke (AIS) patients, due to various reasons, less than 5% of eligible AIS patients received such treatment in the therapeutic window period [2–4]. It seems that due to the delay in referring patients, considerable number of AIS patients lose the chance to receive treatment [5, 6]. Therefore, various interventions have been considered and applied by health care systems during the last decades to reduce the time between AIS onset and starting an appropriate management [7–9]. One of the most important measures was the reduction of wasting time both at the pre-hospital phase and also after admission to the emergency department (ED) [10, 11]. Several stroke screening tools have been designed in this regard that can accurately predict stroke [11], that mostly applied in prehospital setting and their performance has been rarely evaluated in ED environment. Rapid Arterial oCclusion Evaluation (RACE) scale is one of such tools that designed by Pérez de la Ossa et al. not only for screening the stroke cases, but also to differentiate large vessel occlusion (LVO) cases than the others [12]. Despite of high sensitivity and negative predictive value in the primary derivation/validation study, various results were further reported by other investigators about it [13, 14]. It seems that the available data on its performance is still under debate and more focused on differentiating LVO cases and not all AIS patients, and to the best of our knowledge, no one has tested its performance in the ED till yet. We believed that it needs more study to resolve the current controversies, and more importantly, accuracy of RACE for differentiating all AIS cases from the stroke mimics should be evaluated. As a result, we intend to evaluate the accuracy of the RACE criteria in diagnosing of AIS in patients transferred to the ED.

## Methods

### Study design and population

The present study was a diagnostic accuracy cross-sectional study that was conducted from 15 to 2021 until 28 December 2021. The research environment was the ED of research, educational and medical centers in Tehran, Isfahan, and Ahvaz cities, in Iran. The study population was all patients who transferred to the ED by EMS with suspected AIS diagnosis.

According to a study by Pérez de la Ossa et al. [12], assuming a sensitivity of 100% and a specificity of 44%, the prevalence of AIS (patients suspected of stroke transferred to an emergency department) was 45%, estimation accuracy was 95% and error was 5%. The sample size was 841 people.

### Data collection

For this purpose, a checklist consisting of three sections was prepared. The first part related to the basic and demographic information of the patients including age, gender, history of underlying diseases, etc.; the second part consisted of 5 items related to the RACE scale [facial palsy (scored 0–2), arm motor function (0–2), leg motor function (0–2), gaze (0–1), and aphasia or agnosia (0–2); each person can obtain between zero to 9 points]; and the third part included the final diagnosis. The diagnosis of AIS was made based on the findings of physical examination and also interpretation of patients’ brain magnetic resonance imaging (MRI) which is considered as the gold standard method of AIS diagnosis in the current study.

Sampling was conducted prospectively. From the beginning of the study period, the checklist was included in the patients’ hospital files to assess proper neurological examination of patients with any neurological complaints. A postgraduate year (PGY)-2 neurology resident used the RACE scale to evaluate the suspected patients and recorded all documents in the checklist when the patient had been arrived in the ED. A double check on the checklist was also performed by a PGY-3 emergency medicine resident.

### Statistical analysis

The data collected by patient evaluation according to RACE scale was compared with the results of the physician’s diagnosis, as gold standard. We used the ROC curve and the area under the ROC curve (AUC) to evaluate the differentiated power of the RACE scale. The accuracy indices of RACE scale, like sensitivity, specificity, positive and negative predictive value calculated for different cut-off point. To determine the best cutoff point, we used the Youden index and the maximum of vertical distance of ROC curve from the point (x, y) on diagonal line. Also, we used of the univariable and multivariable logistic regression model for assess of effect size of any RACE criteria scale in AIS diagnosis. All statistical analysis conducted with Stata 14 software, College Station, TX: StataCorp LP. The statistical analysis conducted menu-base if available in the software and commands are used to perform ROC analysis, like roctab, dtroc, and cutpt (option: near and youden) syntax. Also, we used of tabulate data for calculate of specifying detail of indices of RACE scale performance for each of the possible cut-points.

## Results

### Baseline findings

In this study, data from 805 patients with suspected AIS transferred to the ED were collected. There were 463 (57.5%) males and 342 (42.5%) females among the studied patients. The mean age ± standard deviation (SD) of the patients was 66.9 ± 13.9 years, and the age range of the patients varied between 6 and 95 years. Table 1 presents some of frequency distribution of risk factors and underline diseases in the study patients.

**Table 1:** Frequency distribution of risk factors and underline diseases in the study patients

Variable	Number (%)	Total	Missing
**Smoking**		number	
Yes	143 (17.8)	803	2
No	660 (82.2)		
**Hypertension**			
Yes	506 (62.9)	804	1
No	298 (37.0)		
**Ischemic heart disease**			
Yes	287 (35.7)	804	1
No	517 (64.3)		
**Diabetes**			
Yes	245 (30.5)	803	2
No	558 (69.5)		
**Coagulopathy**			
Yes	23 (2.9)	802	3
No	779 (97.1)		
**Cerebrovascular accident**			
Yes	127 (15.8)	805	0
No	678 (84.2)		
**Hyperlipidemia**			
Yes	161 (20.0)	803	2
No	642 (80.0)		
**Epilepsy**			
Yes	19 (2.4)	804	1
No	785 (97.6)		

The mean blood glucose level of the patients on arrival to the ED was 148.2 ± 68.1 mg/dL, and its range varied between 27.0 and 560.0 mg/dL. The mean time interval between the onset of symptoms and the arrival at the ED was 8.4 ± 9.9 h and median was 2.5 h, that varied between half an hour to 72 h.

Of all the patients suspected of stroke who transferred to the ED, 562 (69.8%) had a definite final diagnosis of AIS, and the rest of 243 (30.2%) patients were negative for this diagnosis. In other words, the prevalence of AIS in transferred patients was 69.8% (95% CI: 66.6 to 73.0%). Patients with a positive diagnosis of AIS were older than patients with a negative diagnosis. The mean age of the patients with and without AIS was 68.1 ± 13.2 years and 64.1 ± 14.9 years, respectively (p < 0.001). The prevalence of AIS was also higher in men than women (73.9% vs. 64.3%; p = 0.004). Besides that, this prevalence of AIS diagnosis was higher in patients with a history of ischemic heart disease than patients without a similar history (74.9% vs. 67.1%; p = 0.021).

### Detailed results of RACE criteria

Based on the total RACE score, 221 patients (27.5%) had a score of zero. The prevalence of AIS in patients with a zero score was 29.4%, while this value was 100% for patients with a score of 8 and 9 (Table 2).

**Table 2:** Frequency distribution (prevalence) of AIS in the patients with suspected stroke transferred to emergency department by RACE score

RACE score	Total patient for each score	Definite diagnosis of acute ischemic stroke	
		Positive	Negative
		Number (%)	
0	221	65 (29.4)	156 (70.6)
1	31	14 (45.2)	17 (54.8)
2	103	63 (61.2)	40 (38.8)
3	52	45 (86.5)	7 (13.5)
4	97	93 (95.5)	4 (4.1)
5	45	37 (82.2)	8 (17.8)
6	83	77 (92.8)	6 (7.2)
7	78	73 (93.6)	5 (6.4)
8	63	63 (100)	0 (0.0)
9	32	32 (100)	0 (0.0)

Assessment of the relationship between each item of the RACE criterion showed that all items of this criterion have a statistically significant relationship with the final diagnosis of stroke. So that the frequency of disorders in each item was higher in patients who had a definitive diagnosis of AIS than the others (Table 3).

**Table 3:** Frequency distribution and relationship of each RACE criteria with a definitive diagnosis of AIS in the patients with suspected stroke transferred to emergency department (n = 805)

Variable	Total number (%)	Final diagnosis		P-value
		Stroke (n = 562)	Non- stroke (n = 243)	
**Facial Palsy**				< 0.001
Absent (0)	455 (56.5)	248 (44.1)	207 (85.2)	
Mild (1)	82 (10.2)	75 (13.3)	7 (2.9)	
Moderate to severe (2)	268 (33.3)	239 (42.5)	29 (11.9)	
**Arm Motor Function**				< 0.001
Normal to mild (0)	350 (43.4)	144 (25.6)	206 (84.8)	
Moderate (1)	173 (21.5)	151 (26.9)	22 (9.1)	
Severe (2)	282 (35.0)	267 (47.5)	15 (6.2)	
**Leg Motor Function**				< 0.001
Normal to mild (0)	371 (46.1)	167 (29.7)	204 (84.0)	
Moderate (1)	170 (21.1)	149 (26.5)	21 (8.6)	
Severe (2)	264 (32.8)	246 (43.8)	18 (7.4)	
**Head & Gaze Deviation**				< 0.001
Absent (0)	697 (86.6)	456 (81.1)	241 (99.2)	
Present (1)	108 (13.4)	106 (18.9)	2 (0.8)	
**Aphasia or Agnosia**				< 0.001
Normal (0)	354 (44.0)	159 (28.3)	195 (80.2)	
Moderate (1)	222 (27.6)	195 (34.7)	27 (11.1)	
Severe (2)	229 (28.4)	208 (37.0)	21 (8.6)	

### The accuracy of AIS diagnosis, and best diagnostic cut-off points

**Fig. 1:**
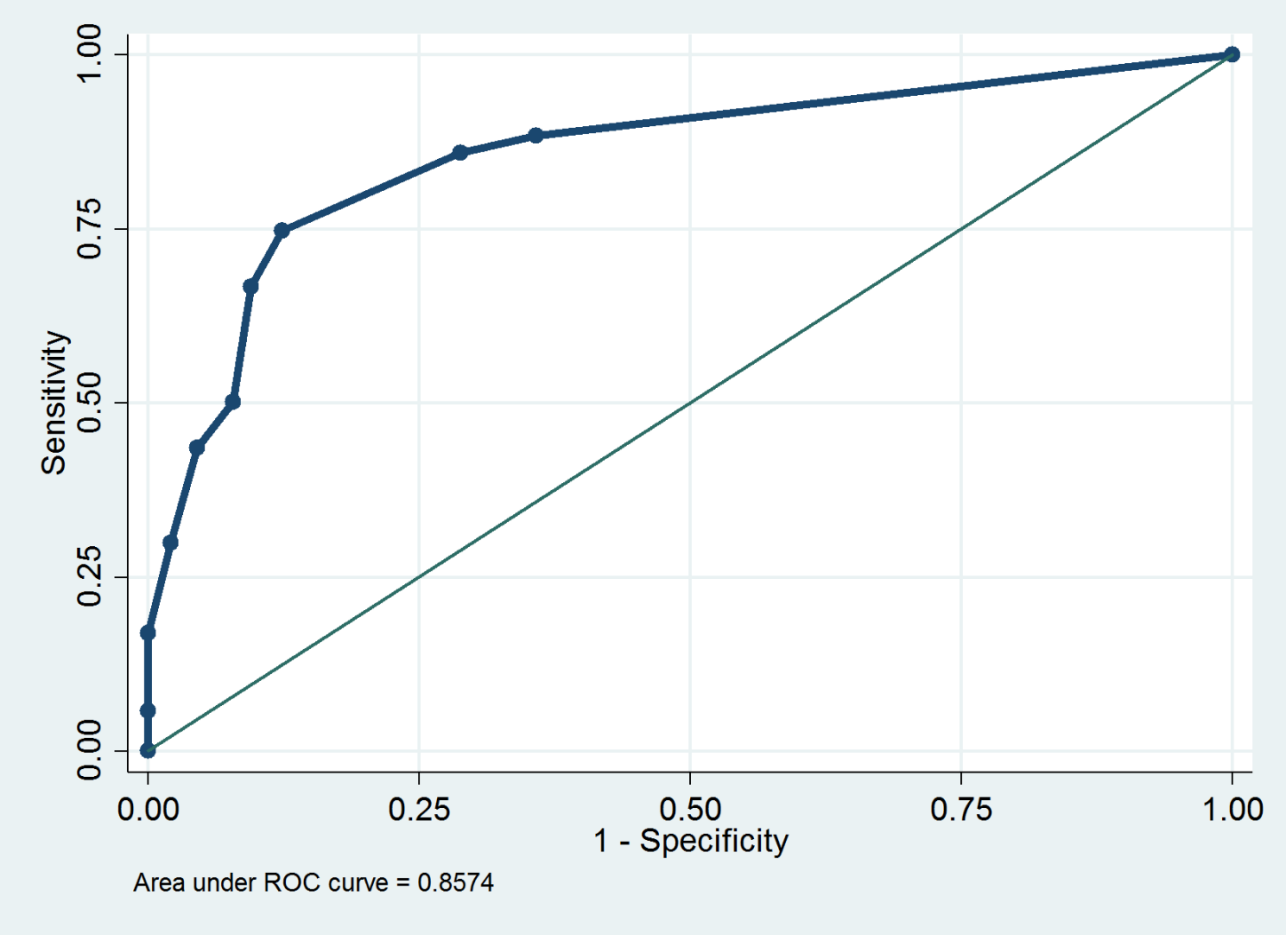
ROC curve for RACE scale in the diagnosis of AIS in patients suspected of stroke transferred to emergency department

The area under the ROC curve of the RACE scale in the diagnosis of AIS was 0.86 (95% confidence interval (CI): 0.83 to 0.88; p < 0.001). (Fig. 1).

The recommended cut-off point for the RACE tool for stroke screening in suspected patients was a score greater than 4. Based on this cut-off point, 62.8% of the patients had an appropriate classification. In other words, at this cut-off, 19 patients (2.4%) were negatively screened for AIS by mistake (false negative), and 280 patients (34.8%) were positively screened for AIS incorrectly (false positive). However, the highest percentage of correct classification (81.5%) and consequently the lowest error in screening according to RACE tool related to the cut-off point greater than one (Table 4).

**Table 4:** Frequency distribution of true and false positives and negatives in the diagnosis of AIS based on various cut-off points of the RACE scale and the percentage of correct classification

Cut-off point	Diagnosis of stroke				Correct classification (%)
	True negative	False negative	True positive	False positive	
	Number (%)				
> 0	156 (19.4)	87 (10.8)	497 (61.7)	65 (8.1)	81.1
> 1	173 (21.5)	70 (8.7)	483 (60.0)	79 (9.8)	81.5
> 2	213 (26.5)	30 (3.7)	420 (52.2)	142 (17.6)	78.7
> 3	220 (27.3)	23 (2.9)	375 (46.6)	187 (23.2)	73.9
> 4	224 (27.8)	19 (2.4)	282 (35.0)	280 (34.8)	62.8
> 5	232 (28.8)	11 (1.4)	245 (30.4)	317 (39.4)	59.2
> 6	238 (29.6)	5 (0.6)	168 (20.9)	394 (48.9)	50.5
> 7	243 (30.2)	0 (0.0)	95 (11.8)	467 (58.0)	42.0
> 8	243 (30.2)	0 (0.0)	32 (4.0)	530 (65.8)	43.2

The sensitivity and specificity of RACE scale for the recommended cut-off point (score > 4) were 50.18% and 92.18%, respectively. According to the Youden J index and maximum of vertical distance, the best cut-off point for this tool was a score greater than 2, at which sensitivity and specificity were 74.73% and 87.65%, respectively. In the best cut-off point the positive and negative predictive values ​​of the RACE tool were 93.3% and 60.0%, respectively (Table 5).

**Table 5:** Accuracy and predictive value indicators for RACE tool at different cut-off points for screening of AIS

Optimal cutoff point	Sensitivity	Specificity	PLR	NLR	PPV	NPV
	Value (95% CI)					
> 0	88.43 (85.5–91.0)	64.20 (57.8–70.2)	2.47 (2.1–2.9)	0.18 (0.1–0.2)	85.1 (82.0–87.9)	70.6 (64.1–76.5)
> 1	85.94 (82.8–88.7)	71.19 (65.1–76.8)	2.98 (2.4–3.6)	0.20 (0.2–0.2)	87.3 (84.3–90.0)	68.7 (62.5–74.3)
> 2*	**74.73 (70.9–78.3**)	**87.65 (82.8–91.5**)	**6.05 (4.3–8.5)**	**0.29 (0.2–0.3**)	**93.3 (90.6–95.5**)	**60.0 (54.7–65.1)**
> 3	66.73 (62.7–70.6)	90.53 (86.1–93.9)	7.05 (4.8–10.4)	0.37 (0.3–0.4)	94.2 (91.5–96.3)	54.1 (49.1–59.0)
> 4	50.18 (46.0–54.4)	92.18 (88.1–95.2)	6.42 (4.1–10.0)	0.54 (0.5–0.6)	93.7 (90.3–96.2)	44.4 (40.1–48.9)
> 5	43.59 (39.4–47.8)	95.47 (92.0–97.7)	9.63 (5.4–17.3)	0.59 (0.5–0.6)	95.7 (92.4–97.8)	42.3 (38.1–46.5)
> 6	29.89 (26.1–33.9)	97.94 (95.3–99.3)	14.53 (6.0–34.9)	0.72 (0.7–0.8)	97.1 (93.4–99.1)	37.7 (33.9–41.6)

*The best cut-off point for stroke screening based on RACE criteria. PLR: positive likelihood ratio; NLR: Negative likelihood ratio; PPV: Positive predictive value; NPV: Negative predictive value; CI: Confidence interval

### Logistic regression results of RACE scale

Logistic regression analysis showed that among the RACE criteria in univariable analysis, all criteria unaccompanied had a significant relationship with AIS diagnosis, to put it another way, the risk of AIS in patients for whom each of the criteria was positive was significantly higher and this relation for the “Head & Gaze Deviation” criterion was the highest, and the risk of AIS in the patients who were positive for this criterion was more than 28 times than that of the patients who were not.

Multivariable regression showed that the variables “Arm Motor Function” and “Head & Gaze Deviation” had the highest and most significant relationship with AIS diagnosis. So, the risk of AIS for the patients with severe “Arm Motor Function” compared to normal ones, regardless of the status of other variables, was 20.6 times higher. The results of multivariable analysis showed that among the RACE criteria, the “Leg Motor Function”, after removing the confounding effect of other variables, had no significant relationship with AIS. Therefore, this criterion has the lowest screening value among other RACE criteria (Table 6).

**Table 6:** Univariable and multivariable logistic regression of RACE criteria in the diagnosis of ischemic stroke in patients suspected of stroke transferred to a hospital emergency department

Variable	Univariable analysis		Multivariable analysis	
	OR (95% CI)	P-value	OR (95% CI)	P-value
**Facial Palsy**				
Mild (1) vs. Absent (0)	8.94 (4.03, 19.8)	< 0.001	1.99 (0.74, 5.30)	0.171
Moderate to severe (2) vs. Absent (0)	6.88 (4.49, 10.55)	< 0.001	4.84 (2.96, 7.90)	< 0.001
**Arm Motor Function**				
Moderate (1) vs. Normal to mild (0)	9.82 (5.98, 16.11)	< 0.001	5.52 (2.41, 12.67)	< 0.001
Severe (2) vs. Normal to mild (0)	25.46 (14.52, 44.67)	< 0.001	20.58 (5.56, 76.21)	< 0.001
**Leg Motor Function**				
Moderate (1) vs. Normal to mild (0)	8.67 (5.25, 14.30)	< 0.001	1.33 (0.56, 3.13)	0.518
Severe (2) vs. Normal to mild (0)	16.70 (9.92, 28.09)	< 0.001	0.30 (0.08, 1.06)	0.062
**Head & Gaze Deviation**				
Present (1) vs. Absent (0)	28.01 (6.86, 114.456)	< 0.001	5.10 (1.15, 22.74)	0.033
**Aphasia or Agnosia**				
Moderate (1) vs. Normal (0)	8.86 (5.63, 13.94)	< 0.001	1.95 (1.08, 3.51)	0.027
Severe (2) vs. Normal (0)	12.15 (7.40, 19.93)	< 0.001	3.43 (1.85, 6.39)	< 0.001

## Discussion

In this study, we assessed the accuracy and other statistic characteristics of the RACE scale in terms of AIS diagnosis for patients with symptoms of stroke referring by EMS to the ED, which showed that the RACE scale is a helpful and accurate diagnostic tool to detect and screen AIS patients. As it was mentioned, the primary derivation/validation study on the RACE scale was performed for differentiated LVO cases, and not all AIS patients [12]. But here, we assessed its accuracy to differentiating, not only LVO cases but also all AIS cases; so the findings should be discussed cautiously, keeping this important issue in mind. Furthermore, in this study, the RACE scale which designed for using in the prehospital settings, was evaluated at the time of the patient arrival in ED. Therefore, we did not examine the validity of the RACE scale as a prehospital scale. Rather, we investigated the performance of this tool for diagnosis of AIS in ED.

The best cut-off point proposed in original article of RACE was score ≥ 5 for differentiating LVO cases [12], but we found that at this cut-off, the criteria had a sensitivity as low as 43.59, and a specificity of 95.47 for differentiating all AIS cases. While we suggest score > 2 as the best cut off for differentiating all AIS cases and improving the sensitivity of the RACE tool that showed a sensitivity of 74.73 and a specificity 87.65 at this cut-off point.

Carrera et al. (that seems to be participated in the primary derivation/validation study on the RACE scale [12]) conducted another two studies on this scale [13, 15]. First they tried to assess predictive value of modified versions of the RACE scale and defined 7 simpler versions of this scale, in this regard; but finally concluded that the original RACE has priority upon all modified versions [13]. Thereafter, they performed another study to revalidation of the RACE scale, in which they confirmed the scale’s accuracy to identify AIS patients with LVO [15]. In both studies by Carrera et al., and also all other conducted studies, the investigators considered the score ≥ 5 as the cut off of their analysis [13–17], and we cannot find any paper, in which somebody tried to assess to find another cut-off with aim of using this test for screening all AIS cases and not just LVO ones.

Thavarajah et al. [18] recently published an article in which they determined that the RACE scale has an acceptable discriminative power for identifying LVO patients in USA, however the reported statistical characteristics was lower than that of reported by the main study conducted in Spain [12] (sensitivity of 0.71 vs. 0.85, specificity of 0.65 vs. 0.68). However, we found even a lower sensitivity, meanwhile, a higher specificity than that of reported by United States-based study [18] and also Spanish-based ones [12, 13, 15].

In a multicenter, prospective, observational cohort study, Duvekot et al. [19] compared 8 prehospital stroke scales to detect LVO cases and reported that of all assessed scales, the AUC for RACE was highest and it was equal to 0·83 (95%CI: 0·79 − 0·86). While, we found that the AUC of the RACE scale in the diagnosis of AIS was 0.86 (95%CI: 0.83–0.88).

The results of logistic regression analysis in this study showed that among the variables of the RACE scale, “Arm Motor Function” and “Head & Gaze Deviation” have a larger effect size than other variables for predicting AIS. In the clinic, the importance of these two variables, or in fact, the findings from the physical examination of patients referred to ED in terms of stroke diagnosis, is very high. These findings are in line with other studies, in which, it has also been claimed that patients who are positive for these variables have a higher chance of having a definite diagnosis of AIS than those patients who are negative for these variables [13, 20].

All in all, we found that, if the RACE would be considered by a health system, as a stablished AIS diagnostic clinical tool, the cut-off > 2 would be more suitable. If the referral stroke centers for AIS cases could be differ based on equipment and availability of thrombectomy facilities, then proposed cut-off ≥ 5 should be considered. But such a facilities are not frequently available in most low and middle-income countries, so it is better to choose a proper clinical tool for stablishing in EMS system, based on each country situation, that can accurately differentiate AIS from none-AIS cases at the first step.

### Limitations

We did not define and categorize the diagnosis of not AIS cases who studied in this survey, and we could not report the stroke mimics that would be valuable. Also, we did not divide the LVO cases from all AIS cases, so we can not exactly validate the defined RACE scale in its original article.

### Future directions

To the best of our knowledge, almost all of previous studies were conducted to evaluate the validity or accuracy of the RACE scale, comparing its statistical indexes with other similar scales, or its performance in real practice for detecting LVO cases. We cannot find any paper, in which somebody tried to assess to find another cut-off with aim of using this test for screening all AIS cases and not just LVO ones. Therefore, we suggest other investigators to consider this point in their future researches.

## Conclusion

It seems that, the RACE scale is an accurate diagnostic tool to detect and screen AIS patients in ED, Of course, not at the previously suggested cut-off point (score ≥ 5), but at the score > 2.

## Electronic supplementary material

Below is the link to the electronic supplementary material.

Supplementary Material 1

## Data Availability

The study used data is available via contacting the corresponding author.

## Abbreviations

AIS: acute ischemic stroke
AUC: area under the ROC curve
CI: confidence interval
ED: emergency department
EMS: emergency medical services
LVO: large vessel occlusion (LVO)
MRI: magnetic resonance imaging
PGY: postgraduate year
RACE: Rapid Arterial oCclusion Evaluation
SD: standard deviation
